# *Mycoplasma hyorhinis* as a possible cause of fibrinopurulent meningitis in pigs? - a case series

**DOI:** 10.1186/s40813-020-00178-8

**Published:** 2020-12-03

**Authors:** Moritz Bünger, Rene Brunthaler, Christine Unterweger, Igor Loncaric, Maximiliane Dippel, Ursula Ruczizka, Lukas Schwarz, Alfred Griessler, Thomas Voglmayr, Doris Verhovsek, Andrea Ladinig, Joachim Spergser

**Affiliations:** 1 University Clinic for Swine, Vetmeduni Vienna, Vienna, Austria; 2 Institute of Pathology, Vetmeduni Vienna, Vienna, Austria; 3 Institute of Microbiology, Vetmeduni Vienna, Vienna, Austria; 4 Traunkreis Vet Clinic, Ried im Traunkreis, Traunkreis, Austria; 5 VetFarm Medau, Vetmeduni Vienna, Berndorf, Austria

**Keywords:** *Mycoplasma hyorhinis*, Meningitis, Central nervous signs, Nursery piglets

## Abstract

**Background:**

*Mycoplasma hyorhinis* is an invader of the upper respiratory tract in swine that is considered to have ubiquitous distribution. It is mainly known for causing polyserositis and polyarthritis in weaned piglets, even though the mechanisms of systemic spread are not fully understood. *Mycoplasma hyorhinis* has also been associated with other diseases in pigs such as pneumonia or otitis media, but so far has not been known to cause central nervous disorders. This case series reports the isolation of *Mycoplasma hyorhinis* from cerebrospinal fluid and/ or meningeal swabs from piglets originating from four different piglet producing farms in Austria.

**Case presentation:**

On farm 1, coughing, stiff movement and central nervous signs occurred in nursery piglets. *Mycoplasma hyorhinis* was the only pathogen isolated from meningeal swabs from two piglets showing central nervous signs. Fibrinopurulent leptomeningitis was only observed in one piglet. Only one of two nursery piglets from farm 2 showed mild central nervous signs but no histologic lesions; *Mycoplasma hyorhinis* was isolated from cerebrospinal fluid of the piglet with neurologic signs. *Mycoplasma hyorhinis* was isolated from cerebrospinal fluid of all three investigated piglets from farm 3, all of which showed central nervous signs and purulent leptomeningitis. Further, *Streptococcus suis* was isolated from the cerebrospinal fluid of one piglet. Fibrinopurulent leptomeningitis was detected in two piglets from farm 4 that had died overnight without showing any clinical signs and *Mycoplasma hyorhinis* was isolated from meningeal swabs from both piglets.

**Conclusion:**

While causality has yet to be proven by experimental infection and in situ detection of the pathogen in histologic sections, the findings of this study and the absence of other pathogens suggest *Mycoplasma hyorhinis* as a potential causative agent of meningitis in swine.

## Background

Since many years, *Mycoplasma hyorhinis* (Mhr) is known as a commensal of the upper airways in swine [1, 2]. It is considered to have ubiquitous distribution and seems to primarily act as a secondary invader [3]. Mhr colonizes the respiratory epithelium and may damage the cilia and reduce epithelial thickness [4]. However, it can also invade joints and serosal tissues after systemic spreading, which mainly originates from colonized mucosal areas. However, exact mechanisms leading to systemic spread are barely understood [5]. Arthritis and polyserositis in pigs 3–10 weeks of age are typical clinical signs of systemic infection; older piglets usually develop mild arthritis [1–3]. Mhr is also implicated in causing conjunctivitis [6], otitis media and eustachitis [7, 8], abortion [9] and lung lesions [10]. Under experimental conditions, Mhr has been shown to induce pneumonia with typical mycoplasma-associated lesions like interstitial pneumonia and perivascular and peribronchial infiltration of lymphoid cells [11]. However, Mhr alone is not considered to cause gross lung lesions or respiratory signs under field conditions [4] but may contribute to the Porcine Respiratory Disease Complex (PRDC) by aggravating pneumonic lesions caused by other primary pathogens like the porcine reproductive and respiratory syndrome virus (PRRSV) [4].

In this case series, we report the isolation of Mhr from meningeal swabs and/or cerebrospinal fluid of piglets with central nervous signs and with or without histopathologic lesions in the brain, originating from four different farms in Austria. Results presented in this case series suggest Mhr to be a potential pathogen of the central nervous system (CNS) in swine. So far, there has been only one previous report on Mhr isolated from the CNS of experimentally infected piglets showing severe arthritis but no central nervous signs [12]. However, our findings suggest including Mhr into the panel of mycoplasma species that have been linked to brain invasion and CNS diseases observed in other animal species and humans [13].

## Case presentation

The piglets of all cases were referred to the University Clinic for Swine, Vetmeduni Vienna, by the responsible herd veterinarian. For this purpose, the herd veterinarian submitted acutely sickened piglets from farms 1 to 3 for necropsy and further diagnostic investigations. Two piglets that had died overnight on farm 4 were transported to the Vetmeduni and necropsied on the consecutive day.

Prior to euthanasia clinical examination was performed by veterinarians of the University Clinic for Swine. Necropsy, the pathomorphologic and the histopathologic examination were performed by the Institute of Pathology, Vetmeduni Vienna. For bacteriological and virologic examinations samples were submitted to the Institute of Microbiology, Vetmeduni Vienna, and the Institute of Virology, Vetmeduni Vienna, respectively. PCR diagnostics for PCV-2 and PRRSV were conducted using routine diagnostic protocols of the Institute of Virology, Vetmeduni Vienna, as previously described [14]. Mhr was diagnosed by cultural isolation from either meningeal swabs (transported in Amies medium) or cerebrospinal fluid using both Friis medium [2] and modified SP4 medium [15]. Briefly, swabs or 100 μl of cerebrospinal fluid were placed into 900 μl of 2SP sample preparation buffer [16], vortexed and 100 μl of suspension were transferred onto Friis and modified SP4 agar incubated at 37 °C in 5% CO_2_ atmosphere for up to 10 days. Plates were checked daily for mycoplasma growth using a stereo microscope. For the specification of mycoplasmas isolated, single colonies were picked, enriched in corresponding broth medium, and identified by MALDI-TOF mass spectrometry (MS) as previously described [17]. For this purpose, the commercially available Bruker taxonomy database was used in conjunction with a large in-house library currently containing 810 mollicutes reference spectra including those generated for type strains and defined clinical isolates of all cultivable porcine mycoplasma species. Using MALDI-TOF MS, all mycoplasma isolates were unambiguously identified as Mhr by producing log score values above 2.00 to Mhr reference spectra whereas log scores to other mollicutes species (and species included in the Bruker taxonomy database) were considerably low, never exceeding values of 1.70. In addition, bacterial samples were examined for other microbes using standard protocols applied for routine bacteriological examinations, including cultivation on Columbia agar with 5% sheep blood and Chocolate II agar incubated at 37 °C in 5% CO_2_ atmosphere or anaerobically (one Columbia agar plate) for up to 72 h. Bacterial colonies isolated were identified by MALDI-TOF MS as previously described [16]. Bacterial and mycoplasma growth was semi-quantitatively graded as scant, light, moderate or heavy. Testing for *Glaesserella parasuis* (GPS) was carried out by PCR as previously described [18].

### Farm 1

In a farrow-to-finish farm with 80 sows and 350 fattening pigs, 20–30% of the piglets in the nursery were coughing, wasted, had stiff movement and about 1% showed central nervous signs like head tilt and locomotive disorders. The mortality rate in nursery pigs was 5%. At the University Clinic for Swine, Vetmeduni Vienna, two piglets with central nervous signs at the age of 8 weeks were euthanized and necropsied. Piglet A arrived in lateral recumbency and was not able to move, had swollen joints and was coughing. Piglet B was stiff while moving with swollen tarsal joints and showed head tilt. During necropsy, a yellowish ascites fluid (piglet B) as well as fibrin strands (both piglets) were seen in the abdomen. The tarsal joints of piglet A were swollen with an increased amount of synovia including fibrinous fluid, though the cartilage did not show any macroscopically discernible abnormalities and no inflammation of the synovial membranes were obvious. Additionally, the meninges of piglet B were turbid. Histopathologic lesions in both piglets included fibrinopurulent pleuritis, severe fibrinopurulent epicarditis and moderate to severe fibrinopurulent peritonitis. In both piglets, interstitial, intralobular and peribronchial pneumonia was observed while type II pneumocytes were proliferated. During histopathologic examination of the meninges of piglet B, severe leptomeningitis, mainly lymhocytic and partly fibrinopurulent, was detected.

Lung tissue, serosal swabs, meningeal swabs, and synovia from both piglets were sampled for bacteriological examination. Except for some secondary invaders in the lung (*Bordetella* (*B.*) *bronchiseptica* (piglet B) and *Staphylococcus* (*Staph.*) *aureus* (piglet A)), Mhr was the only agent isolated (moderate to heavy grade) from samples taken including meningeal swabs. All samples were also negatively tested for GPS by PCR. A pool consisting of lung tissue, tracheobronchial lymph node and tonsil was tested positive for PRRSV by Open Reading Frame 7 (ORF7) RT-PCR in both piglets, turning out to be a PRRSV-1 subtype 1 isolate by ORF5 sequencing. Investigating inguinal lymph node tissues for viral load of Porcine Circovirus 2 (PCV-2) by quantitative PCR (q-PCR) yielded positive results for both piglets that were below the limit of quantification (< 1 × 10^4^ GE/mg) [14]. The combination of PRRS stabilization using a modified life virus (MLV) vaccine and a single dose of tulathromycin at weaning seemed to be successful in decreasing the losses to 1% and eliminating central nervous signs in piglets after weaning.

### Farm 2

In a farrowing farm with 50 sows and a nursery unit with straw bedding and without an All-In/All-Out production, piglets showed respiratory signs beginning at the age of seven to 8 weeks. The farm was known to be PRRSV positive and sows were vaccinated three times a year using MLV vaccine. At the University Clinic for Swine, Vetmeduni Vienna, two nursery piglets were euthanized and necropsied. During clinical examination prior to euthanasia, respiratory signs were observed in both piglet A (7.50 kg) and piglet B (15.00 kg), while head tilt and signs of wasting were present only in piglet A.

During necropsy, severe adhesive epicarditis and pleuritis, moderate to severe fibrinopurulent peritonitis and severe pneumonia of mainly the cranial lobes were seen in both piglets. In the lungs of both piglets, moderate to severe intralobular, interstitial bronchopneumonia with beginning (piglet B) or severe (piglet A) fibrosis was histopathologically discernible. In piglet A, histopathologic examination additionally revealed severe inflammatory infiltration with round cells of the mucous membrane of the stomach, as well as a purulent inflammation of the right bulla tympanica with beginning osteolysis of the surrounding bones.

Serosal swabs and lung tissue from both piglets were sampled for bacteriological examination. Cerebrospinal fluid was only sampled from piglet A, as piglet B did not show any central nervous signs. Serosal swabs from both piglets and the cerebrospinal fluid from piglet A were negative for GPS by PCR. *Mycoplasma hyopneumoniae* was detected by PCR in the lung tissue of both pigs. Mhr was successfully cultivated from serosal swabs of both piglets (heavy grade) and the cerebrospinal fluid of piglet A (heavy grade). From the lung tissue of piglet A, *Streptococcus* (*S.*) *suis* and *B. bronchiseptica* were cultivated (light grade), as was *S. suis* in the serosal swab (light grade). In piglet B, *S. orisratti, Staph. aureus* and hemolytic *Escherichia* (*E.*) *coli* were found in lung tissue and serosal swabs only after enrichment. In both piglets, PCV-2 was negative by q-PCR from lymph node tissue, while a pool consisting of lung tissue, tracheobronchial lymph node and tonsils was positive in both piglets for PRRSV by ORF7 RT-PCR. Due to these results, the use of an MLV vaccine had been expanded to nursery piglets for PRRS stabilization in this herd. According to the herd veterinarian, the situation on farm has since improved but is still not optimal, primarily due to management issues. For example, PRRSV vaccination of the weaned piglets was stopped by the farmer due to economic reasons.

### Farm 3

In an Austrian farrow-to-finish farm (320 sows), high fever and late-term abortions as well as increased return to estrus rates were observed in sows. The farm had previously been PRRSV-1 positive but stable without obvious clinical signs and sows were also tested negative for influenza. This outbreak was followed by occurrence of severe problems in weaned piglets, characterized by arthritis (4% of piglets), central nervous signs (2% of piglets) and increased losses. Piglets showed arthritis and coughing (up to 50% of piglets) starting at the age of 3–4 weeks. Mortality of piglets with central nervous signs was 100%, even after treatment. To reduce clinical signs, PRRSV vaccination of sows and later also piglets was established using MLV vaccine.

**Fig. 1:**
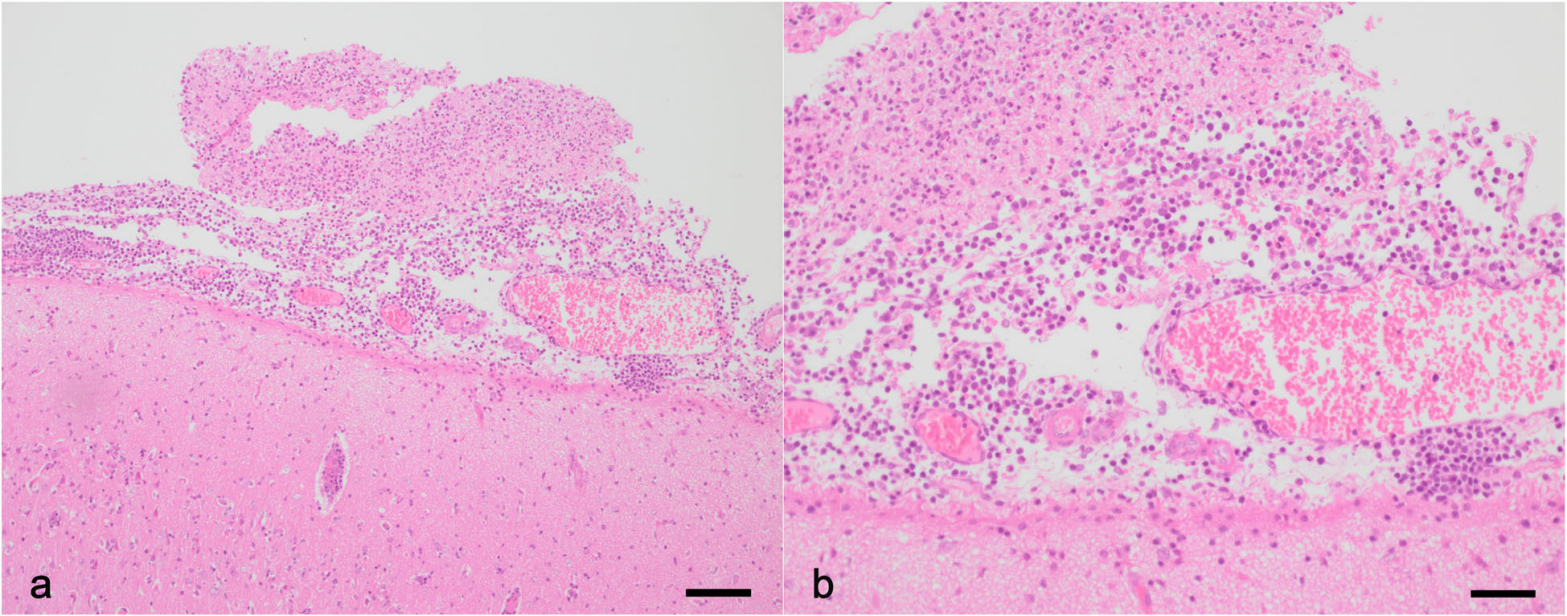
**a + b** Histologic lesions of the brain. Histologic section of the cerebellum of a piglet from farm 3, showing fibrinopurulent meningitis with an increased amount of histiocytes; H&E staining; bar length 100 μm **a** and 50 μm **b**

Three piglets with a body weight around 9 kg were submitted to the University Clinic for Swine, Vetmeduni Vienna, for euthanasia and necropsy. Piglets A and B had previously been treated with Cefquinome and all three of them had been vaccinated with a PRRSV MLV vaccine 3 weeks prior to the necropsy. All three piglets showed moderately (piglets B and C) or severely (piglet A) decreased general behavior, poor body condition and signs of polyarthritis like increasingly filled and warm tarsal and carpal joints. Piglet C showed head tilt, uncoordinated movement and moderately accentuated breathing sound; piglet B showed severe lameness together with decreased responsiveness; piglet A was in lateral recumbency without being able to rise and had ear tip necrosis. During necropsy either mild (piglets B and C) or severe (piglet A) fibrinous peritonitis was observed in all piglets. Tarsal and carpal joints were increasingly filled with cloudy synovia and fibrin strands. The lungs, especially the cranial lobes and the cranial parts of the main lobes, showed signs of pneumonia like atelectasis, hyperemia, and emphysema. Histopathologically, peribronchial, peribronchiolar and perivascular infiltration of round cells was observed in the lungs of all piglets; in the lungs of piglets A and C, purulent bronchopneumonia was also observed. Histopathologic examination of the brain revealed fibrinopurulent leptomeningitis (moderate to severe grade) with an increased amount of histiocytes as well as localized areas with encephalitis in all piglets (Fig. 1). Additionally, moderate purulent meningitis was observed in the spinal cord of piglet A, while only mild signs of inflammation were detectable in the spinal cords of piglets B and C. Inguinal lymph nodes from all three piglets were negative for PCV-2 by qPCR. Peritoneal swabs, cerebrospinal fluid, synovia, lung tissue and the small intestine from all three piglets were sampled for bacteriological examination. Synovia, cerebrospinal fluid and peritoneal swabs were negative for GPS by PCR; cultivation of Mhr from the cerebrospinal fluid resulted in growth of single colonies (scant grade) for piglets A and B and a heavy growth for piglet C. While no further noteworthy bacterial pathogens were found in samples of piglets B and C, *S. suis* was cultivated from the synovia, cerebrospinal fluid, and peritoneal swab of piglet A, as well as *B. bronchiseptica* and *S. suis* from lung tissue. Vaccinating pregnant sows with an autogenous vaccine against *S. suis* and Mhr to stimulate the production of maternal antibodies successfully reduced the occurrence of arthritis and central nervous signs in weaned piglets in this herd.

### Farm 4

In an Austrian farrowing farm with 85 sows and gilts occasional deaths without any clinical signs occurred in suckling piglets. As there had been similar acute deaths in a prior group, the herd veterinarian decided to perform a necropsy on one piglet; fibrin strands, mild ascites and mild pericardial effusion were seen in this piglet. Two 30 day old, recently weaned piglets (both 5.20 kg) that died within a few hours without prior signs were transferred to the University Clinic for Swine, Vetmeduni Vienna, for further investigations. Both piglets had less than good body condition, but no signs of a disease were externally discernible. During pathologic examination fibrinopurulent pleuritis, peritonitis and pericarditis were observed in both piglets, as were hyperemia and atelectasis in the cranial lobes of the lung; histopathologic examination of the cranial lobes revealed focal purulent bronchopneumonia in piglet A and alveolar histiocytosis in piglet B. In both piglets, severe fibrinopurulent meningitis was seen during histopathologic examination of the brains; a purulent and partially abscessing lymphadenitis was only observed in one lymph node from piglet A. Inguinal lymph nodes from both piglets were negative for PCV-2 by qPCR. For bacterial examination serosal swabs, lung tissue and meningeal swabs were sampled from both piglets and a pericardial swab was additionally sampled from piglet A. In piglet A, *Mannheimia haemolytica* was found in samples of lungs, pericardium and pleura; *Staph. aureus* and *Moraxella* sp. were found in the abscessing lymph node and pleura, respectively and single colonies of Mhr were cultivated from the lungs and meningeal swabs. In piglet B, the routine bacterial examination was negative after enrichment, only Mhr was found in lungs (light grade) and meningeal swabs (moderate grade). No further occurrence of acute deaths or other inexplicable clinical signs were observed in that farm ever since.

The described cases took place in the years 2016 (farm 1), 2017 (farm 2) and 2019 (farms 3 and 4). Farm 4 is distantly located from farms 1–3 in a region with a low density of pig farms. Farms 1, 2 and 3 are piglet producing farms located in the same region with high pig density, which in theory could indicate an epidemiological connection between these farms. However, whole genome sequencing and core genome Multi-Locus Sequence Typing (cgMLST) of Mhr isolates from each of these three farms revealed that isolates are only distantly related excluding an epidemiologic relationship between the cases presented in our study (unpublished data).

## Discussion

Previously, there had been only one report on Mhr colonizing the CNS [12] and Mhr has not yet been known to cause central nervous disorders in swine. As this is only the second report of cultivating Mhr from the porcine CNS, the mechanisms of Mhr invading the CNS are completely unknown but could be similar to those for systemic spread to serosal and synovial tissues [1, 2], which are also poorly understood. However, direct entry of Mhr from the nasopharyngeal mucosa to the brain, e.g. via the auditory pathway cannot be excluded.

The study did not determine whether Mhr is only colonizing the CNS or is also acting as a pathogenic agent. Nevertheless, all eight piglets from which Mhr was successfully and, to some extent, abundantly cultivated from cerebrospinal fluid or meningeal swabs, either showed central nervous signs or histopathologic lesions, or both (Table 1). Only piglet B from farm 2 did not show any central nervous signs or histopathologic lesions and no cerebrospinal fluid or meningeal swabs had been examined. In six piglets fibrinopurulent or purulent leptomeningitis suggested an involvement of bacterial pathogens. No bacterial pathogens were isolated from CNS samples of five piglets, while *S. suis*, a well-known and common cause of meningitis in swine [19], was found in one piglet (farm 3, piglet A) even though it had been treated with Cefquinome, as was a second piglet from the same farm (farm 3, piglet B). This leaves Mhr as the only detectable bacterial agent in five of six piglets with histopathologic damage in the brain, including the four piglets that had not been treated with antibiotics, proposing causal involvement in inducing these lesions. However, detection and localization of Mhr using staining methods such as in situ hybridization are required to demonstrate an association of Mhr with histopathologic lesions but have yet to be established and validated in our hands. Finally, reproducing the clinical signs and/or histologic lesions after experimental infection is essential for final prove of Mhr as a causative agent of meningitis in swine.

**Table 1:** Overview of clinical signs, pathohistologic and diagnostics findings

Farm	Piglet	CNS-related clinical signs	Pathohistologic lesions of the brain	Mhr isolation from different tissues	other bacteria isolated from CNS samples	PRRSV status of farms and PCR results of piglets	PCV-2 PCR results of piglets
1	A	lateral recumbency, reduced general behavior	none	serosa +++ meninges +/++ synovia +++	–	PRRSV detected in tissue samples by RT-PCR	below limit of quantification
	B	head tilt	severe lymphocytic and fibrinopurulent leptomeningitis	serosa +++ meninges +/++ synovia +++	–	PRRSV detected in tissue samples by RT-PCR	below limit of quantification
2	A	head tilt, reduced general behavior	none	CSF +++ serosa +++	–	PRRSV detected in tissue samples by RT-PCR	negative
	B	none	none	serosa +++	no samples taken	PRRSV detected in tissue samples by RT-PCR	negative
3	A	lateral recumbency, unable to rise	severe fibrinopurulent leptomeningitis	CSF (+) lung +++ synovia (+)	CSF: *S. suis* +	MLV vaccinated, not tested	negative
	B	uncoordinated movement, lameness, tremor/shaking	moderate fibrinopurulent leptomeningitis	CSF (+)	–	MLV vaccinated, not tested	negative
	C	uncoordinated movement, lameness, tremor/shaking	severe fibrinopurulent leptomeningitis	CSF ++/+++ lung tissue (+)	–	MLV vaccinated, not tested	negative
4	A	peracute deaths, no symptoms	severe fibrinopurulent leptomeningitis	meninges (+) lung (+)	–	unsuspicious, not tested	negative
	B	peracute deaths, no symptoms	severe fibrinopurulent leptomeningitis	meninges ++ lung +	–	unsuspicious, not tested	negative

*Abbreviations*: *CNS* central nervous system, *Mhr Mycoplasma hyorhinis*, *PRRSV* procine reproductive and respiratory syndrome virus, *PCV2* porcine circovirus 2, *CSF* cerebrospinal fluid. *MLV* modified life virus, *S. Streptococcus, −* negative, *(+)* scant growth, *+* light growth, *++* moderate growth, *+++* heavy growth

*S. suis* and GPS are the most common invaders of the CNS and cause of meningitis in swine comparable to the findings in our cases [19]. Both pathogens cross the blood-cerebrospinal fluid barrier (B-CSFB) or blood-brain barrier (BBB) by invading the porcine brain microvascular endothelial cells (PBMECs) [20, 21] which are part of the BBB [22]. They both also increase the permeability of the BBB by inducing the release of IL-8 [23, 24], a feature that Mhr is also known to be capable of since varying levels of IL-8 release have been determined when porcine bone-marrow-derived dendritic cells were stimulated by different Mhr strains [25]. This might suggest that the capability of invading the CNS might differ between strains of Mhr, depending on their ability to induce IL-8 release by currently unknown virulence factors. For *S. suis* and GPS, invasion depends on the activity of particular virulence factors and appears to be associated with certain serotypes [20, 21]. For example, in *S. suis* serotype 2 enolase was suggested to be a potential virulence factor for CNS invasion as it induced IL-8 release [23]. Although enolase, an enzyme of the glycolytic pathway and prototypic moonlighting protein, has shown to act as adhesin on the cell surface of several mycoplasma species [26–30], its potential moonlighting activity in Mhr remains to be elucidated. Interestingly, enolase is also suggested to play a role in human tic disorders and to act as an autoantigen in Hashimoto’s encephalopathy [31, 32]. Neuraminidase/sialidase enzymatic activity has further been suggested as a potential virulence factor for CNS invasion in certain mycoplasma species including *M. neurolyticum*, *M. synoviae* and *M. alligatoris* [13]. Although all genome sequenced Mhr strains possess a sialic acid scavenging and degradation pathway, differences in their potential to cross mucosal barriers and to spread to and invade serosal, synovial and CNS tissues might be explained by a variable expression of sialidase activity as yet observed in other mycoplasmas [33].

Altogether, factors and mechanisms contributing to brain invasion of Mhr and thereby causing CNS disease are largely elusive and remain speculative. As a first step, comparative genome studies of Mhr isolated from different body sites would possibly gain insights into the characteristics defining tissue tropism.

Predisposing factors could further influence the ability of Mhr to systemically spread and, in consequence, invade the CNS. As PRRSV is known to promote *S. suis* septicemia [34], PRRSV could be such a predisposing factor for Mhr as well. However, there currently are no studies that clearly indicate whether PRRSV enhances systemic spread of Mhr and it is unlikely that PRRSV is a definite requirement for invasion of Mhr into the CNS as farm 4 is, and always has been, unsuspicious for PRRS. PCV-2 also does not seem to play any role in the pathogenesis of our four cases, as our findings from farm 1 only suggested a subclinical infection while the piglets of the other three farms were negative for PCV-2.

## Conclusion

It remains unknown whether Mhr is an emerging invader of the CNS or simply has previously not been investigated. The latter seems more likely, as it has already been cultivated from the CNS almost 50 years ago [12] and mycoplasma diagnostics are probably not frequently performed from cerebrospinal fluid or meningeal swabs. This case series suggests that Mhr might not only be an invader of the CNS but also a possible cause of meningitis in pigs, although experimental infections in conjunction with a validated detection method for Mhr in histologic sections like in situ hybridization or RNA scope are required to prove causality. Further, basic research on the pathomechanisms and virulence factors of Mhr is needed, as current knowledge is limited.

## Data Availability

All data generated or analysed during this study are included in this published article.

## Abbreviations

*B.*: *Bordetella*
BBB: Blood-brain barrier
B-CSFB: Blood-cerebrospinal fluid barrier
CNS: Central nervous system
*E.*: *Escherichia coli*
GPS: *Glaesserella parasuis*
Mhr: *Mycoplasma hyorhinis*
MLV: Modified live virus
ORF: Open Reading Frame
PCV-2: Porcine Circovirus 2
PRDC: Porcine Respiratory Disease Complex
PRRSV: Porcine reproductive and respiratory syndrome virus
q-PCR: Quantitative PCR
*S.*: *Streptococcus*
*Staph*: *Staphylococcus*
